# CD4^+^T Cells: Differentiation and Functions

**DOI:** 10.1155/2012/925135

**Published:** 2012-03-14

**Authors:** Rishi Vishal Luckheeram, Rui Zhou, Asha Devi Verma, Bing Xia

**Affiliations:** ^1^Department of Gastroenterology, Zhongnan Hospital, Wuhan University School of Medicine, Wuhan 430071, China; ^2^Center for Clinical Study of Intestinal Diseases, Zhongnan Hospital, Wuhan University School of Medicine, Wuhan 430071, China; ^3^Key Laboratory of Allergy and Immune-Related Diseases, Wuhan University School of Medicine, Wuhan 430071, China; ^4^Department of Paediatrics, Renmin Hospital, Wuhan University School of Medicine, Wuhan 430071, China; ^5^Clinical Centre of Intestinal and Colorectal diseases, Hubei, Wuhan 430071, China

## Abstract

CD4^+^T cells are crucial in achieving a regulated effective immune response to pathogens. Naive CD4^+^T cells are activated after interaction with antigen-MHC complex and differentiate into specific subtypes depending mainly on the cytokine milieu of the microenvironment. Besides the classical T-helper 1 and T-helper 2, other subsets have been identified, including T-helper 17, regulatory T cell, follicular helper T cell, and T-helper 9, each with a characteristic cytokine profile. For a particular phenotype to be differentiated, a set of cytokine signaling pathways coupled with activation of lineage-specific transcription factors and epigenetic modifications at appropriate genes are required. The effector functions of these cells are mediated by the cytokines secreted by the differentiated cells. This paper will focus on the cytokine-signaling and the network of transcription factors responsible for the differentiation of naive CD4^+^T cells.

## 1. Introduction

The human immune system consists of the ancient innate immune system passed on along the evolution from invertebrates and the recently acquired adaptive immune system uniquely present in vertebrates. The principal functions of the immune system are the recognition with subsequent elimination of foreign antigens, formation of immunologic memory, and development of tolerance to self-antigens. The lymphocyte population is mainly made up of the thymus-derived lymphocytes (T-lymphocytes), bone-marrow-derived (B-lymphocytes), and the natural-killer cells (NK cells). T-lymphocytes mediating the cellular immunity, along with B lymphocytes mediating humoral immunity, provide adaptive immunity, which work in close collaboration with the innate immune system. B-lymphocytes mature in the bone marrow itself, while the T-lymphocytes require the thymus to mature, before being deployed to the peripheral lymphoid organs for further antigen-mediated differentiation. A small subset of the CD4^+^cells, including natural regulatory cells and natural killer T cells (NKT cells), are already distinct differentiated cells on release from the thymus.

 CD4^+^T cells along with CD8^+^T cells make up the majority of T-lymphocytes. CD4^+^T cells after being activated and differentiated into distinct effector subtypes play a major role in mediating immune response through the secretion of specific cytokines. The CD4^+^T cells carry out multiple functions, ranging from activation of the cells of the innate immune system, B-lymphocytes, cytotoxic T cells, as well as nonimmune cells, and also play critical role in the suppression of immune reaction. Continuing studies identified new subsets of CD4^+^ cells besides the classical T-helper 1 (Th1) and T-helper 2 (Th2) cells. These include T-helper 17 (Th17), follicular helper T cell (Tfh), induced T-regulatory cells (iTreg), and the regulatory type 1 cells (Tr1) as well as the potentially distinct T-helper 9 (Th9). The differentiation of the different lineages depends on the complex network of specific cytokine signaling and transcription factors followed by epigenetic modifications. This paper will be focusing on the cytokine milieu and lineage specific transcription factors required for the differential development of the antigen-activated CD4^+^T cells, and also will cover a brief overview of the development pathway of mature naïve CD4^+^T cells, and finally the effector functions of each subtype will be summarized.

## 2. Lymphopoiesis

T cells precursors originating from a common lymphoid hematopoietic stem cell leave the bone marrow to reach the thymus for maturation. Initially thought to be an evolutionary remnant with negligible function, the thymus is in fact a primary lymphoid organ indispensable for T-lymphocyte development. The thymus provides a suitable microenvironment with specific combination of stromal cells, cytokines and chemokines to generate functional T cells from T-cell precursors (thymocytes). T-cell receptor (TCR) gene rearrangement and thymocyte selection are the critical steps in the development of mature T-lymphocytes capable of recognizing an infinite range of antigens. During the differentiation process, the migration of thymocytes through discrete thymic microenvironments and contact with peptide-MHC complex (pMHC) on distinct thymic antigen-presenting cells (APCs), including the cortical thymic epithelial cells (cTECs), medullary thymic epithelial cells (mTECs), and dendritic cells (DCs), play a pivotal role in the shaping of the T cell repertoire for antigen recognition, the selection process, and the expression of surface molecules such as CD4 and CD8 [1–3]. The selection process can be depicted by the affinity model, whereby the thymocytes expressing TCR with negligible affinity to pMHC die and those with very high affinity are destroyed (negative selection). Only thymocytes with TCR of intermediate affinity to pMHC undergo positive selection and further differentiation into mainly CD4^+^ and CD8^+^ mature T-lymphocytes [1, 4]. TCR consists of *αβ* or *γδ* chains bonded with five CD3 subunits (*γ*, *δ*, *μ*, *π*, and Σ). TCR interacts with antigen-MHC complex, while CD3 mediates T-cell activation signals [5]. TCR *α* chain is encoded on chromosome 14 and consists of V (variable) and J (joining) genes. The *β* chain genes are located on the 7 chromosome with V, J, and D (diversity) gene segments. The *γ* chain is on chromosome 7, and the *δ* chain on chromosome 14. A vast repertoire of TCR *αβ* is generated by gene rearrangement between exons of the variable domains of the V-J segments of *α* chain and V-D-J segments of *β* chain [6]. Moreover junctional diversity V-N-J, V-N-D, and D-N-J are produced by random insertions/deletions at these regions [7, 8]. The diversity is expressed in the complementary determining regions (CDRs), that make up the antigen-recognition site of the TCR. The T-cell precursor, that is the double positive CD4^+^CD8^+^ thymocyte, differentiates into several mature T cell lineage. Based on the interaction of CD4^+^CD8^+^ cell TCR with pMHC I or II, some nonconventional lineages are also produced along with the classical naïve CD4^+^CD8-T cells and CD4^−^CD8^+^ T cells. The CD4^+^ expressing non-conventional T cells include the FOXP3^+^CD4^+^CD25^+^ natural T-regulatory cells (nTreg cells), and the CD1d-reactive natural killer T (NKT) cells, whereas the CD8^+^ ones are the MHC1b CD8^+^T cells, and the major histocompatibility molecule-related 1(MR1)-restricted mucosa-associated invariant T cells [9]. The NKT cells can be CD4^+^ or CD4^−^CD8^−^. Mature naïve CD4^+^ T cells are then deployed to secondary lymphoid organs, including the spleen, lymph nodes, and the mucosa-associated lymphoid tissue, where they constantly survey for pMHC II molecules, for antigen recognition [10].

## 3. CD4^+^T Cells Activation and Differentiation

The initial step of differentiation of the naïve cells is the antigenic stimulation as a result of interaction of TCR and CD4 as co-receptor with antigen-MHC II complex, presented by professional antigen presenting cells (APCs). TCR coupled with CD3 activation consequently induces a network of downstream signaling pathways, that eventually lead to naïve cell proliferation and differentiation into specific effector cells. Lineage-specific differentiation depends on the cytokine milieu of the microenvironment, as well as on the concentration of antigens, type of APCs, and costimulatory molecules [11, 12]. Among the APCs, the dendritic cells (DCs) are considered to be most important due to their enhanced ability to stimulate naïve T cells [13]. Dendritic cells are activated through the recognition of pathogenic antigens by cell surface pattern recognition receptors, such as toll-like receptor and intracellular pathogen sensing receptors such as the nucleotide oligomerization domain (NOD)-like receptors [14, 15]. DCs consist of different subsets which interfere with the differentiation lineage. In mice, CD8*α* ^+^ DC were involved with Th1 lineage, while the CD8*α* ^−^ subsets were linked to Th2 differentiation, through the secretion of IL-12 and IL-6, respectively [16]. Costimulatory signals augment TCR signals, thereby promoting proliferation and differentiation. The main co-stimulatory receptor is CD28, which is expressed in all naïve T cells. The ligands of CD28 on the DC are the CD80 (B7-1) and CD86 (B7-2), which are upregulated upon activation of DC. Other less potent co-stimulatory molecules include CD28 homolog inducible co-stimulator (ICOS), members of TNF receptor family (CD27, 4-1BB, and OX-40). These receptors have their ligands expressed on DC [17, 18]. The initial source of cytokines are from the APCs as well as other members of the innate immune cells. Subsequently, some of the cytokines produced by the differentiating cells can create a positive feedback loop, whereby the differentiation and response are marginally enhanced.

### 3.1. Th1 Differentiation

Interleukin 12 (IL12) and interferon *γ* (IFN*γ*) are the critical cytokines initiating the downstream signaling cascade to develop Th1 cells [19]. IL12 is secreted in large amounts by APCs after their activation through the pattern recognition receptors [14, 15, 20]. The IL12, in turn, induces natural killer cells(NK) to produce IFN*γ*.

Several transcription factors in coordination induce full differentiation of the Th1 cells (Table 1). The master regulator for Th1 differentiation, the T-box transcription factor (T-bet), is defined not only by its ability to activate the set of genes to promote differentiation of a particular phenotype, but also by that of being able to suppress the development of opposing cell lineages [21, 22]. T-bet is the principal transcription factor, as it significantly enhances the production of IFN*γ*, and plays important role in suppressing the development of Th2 and Th17 [22, 23]. T-bet expression was found to be strongly dependent on signal transducer and activator of transcription 1 (STAT1), rather than on IL12–dependent STAT4 [21, 24]. STAT1, is in turn activated by IFN*γ*. T-bet further induces IFN*γ* production by the differentiating cells, thereby amplifying T-bet expression and upregulating the expression of IL12R*β*2. The latter cells can then be selected by the abundant IL12 from the APCs, thus ensuring selective expansion of the differentiating Th1 cells [21]. T-bet suppresses development of Th2 cell by inhibiting the crucial IL4 gene and impairing the function of the Th2 master regulator GATA3 [25, 26]. Th17 lineage is inhibited by the interaction of T-bet with Rorc promoter, which encodes ROR*γ*t, the principal transcription factor of Th17 [23].

IL12-induced STAT4 is another important transcription factor involved in the Th1 cell differentiation [27]. STAT4 induces IFN*γ* production, thereby creating a positive feedback loop for further T-bet and IL12R*β*2 expression. STAT4 and T-bet are involved directly in the transcription of IFN*γ* locus through the creation of activating marks at the locus, while STAT6 and GATA3 in Th2 differentiation establish repressive histone marks at the said locus, thereby indicating that the activation of IFN*γ* locus dictates Th1 differentiation [28]. However, STAT4 and T-bet do not function in a linear way in the differentiation of Th1 cell, with each having their unique signaling pathway. But for complete Th1 cell differentiation, these-lineage specific transcription factors need to operate in coordination with one another [29]. In later stages of differentiation, IL12/STAT4 pathway upregulates IL-18R*α*. IL12 along with IL18 induces IFN*γ* production independent of TCR activation, thus creating a pathway for enhancing Th1 response.

Runt-related transcription factors also participate in the differentiation process. Runx1 and Runx3 were found to promote Th1 cell differentiation [16, 25, 30]. Runx3, in coordination with T-bet, binds to the IFN*γ* promoter and silences the genes encoding IL4, leading to the Th1 lineage differentiation [25]. Moreover, Runx3, through interaction with GATA3, leads to the inhibition of Th2 differentiation [16]. Runx1 together with T-bet inhibits Th17 development by interfering with the ROR*γ*t master regulator [23].

Recent studies identified a novel role of T-bet as a transcriptional repressor. T-bet through the induction of transcriptional repressor, Bcl-6, represses the activity of IFN*γ* locus in later stages of Th1 differentiation, with the consequence of reducing the overproduction of IFN*γ* and hence acts as a protective mechanism to avoid immunopathology [31].

Eomesodermin (Eomes), also a member of the T-box gene family, is important in regulating CD8^+^ cells development and functions, and also plays a role in the Th1 lineage commitment. IL 21 represses Eomes expression. Exposure of naïve cell to IL21 led to the reduction of IFN*γ* production by the developing Th1 cells [32].

Hlx, another transcription factor induced downstream to T-bet activation, has been found to enhance IFN*γ* production by Th1 cells [33].

### 3.2. Th2 Differentiation

IL4 and IL2 are critical for Th2 differentiation. The major transcription factor involved in Th2 lineage differentiation includes the IL4-induced STAT6, which upregulates the expression of the master regulator GATA3 (GATA-binding protein) [34–36]. 3 distinct mechanisms of GATA3 involvement in Th2 differentiation have been postulated, including enhanced Th2 cytokine production, selective proliferation of Th2 cells through recruitment of Gfi-1, and inhibition of Th1 differentiation presumably by interacting with T-bet [37]. Moreover, GATA3 was found to suppress Th1 differentiation by downregulating STAT4 [38]. In vivo, GATA3 is indispensable for Th2 response. In GATA3 deficient mice, differentiation of naïve cells was diverted towards the Th1 lineage [39]. Absence of GATA3 leads to the interruption of Th2 differentiation [37, 39, 40]. Recent studies showed that GATA3 by itself cannot regulate all the Th2-specific genes, but instead needed the collaboration of STAT6 [41]. Although IL4 and IL2 are required for Th2 cells development in vitro, there is evidence of IL4-independent Th2 differentiation in vivo. But since GATA3 is indispensable for Th2 cells differentiation in vivo, it can be suggested that there exist an IL4-independent GATA3 activation pathway [42, 43]. Continuing researches showed that Th2 cell differentiation involves several other transcriptional factors activated downstream to several cytokines, including IL2, IL6, and IL21.

STAT5 has an important role in the Th2 lineage commitment. It is readily activated by IL2 [44, 45]. STAT5 activation is independent of IL4 signaling and does not induce GATA3 expression [46]. For full differentiation of Th2 cells, the coordinated activity of STAT5 and GATA3 is required, since GATA3 alone cannot induce the production of IL4. This is due to the fact that GATA3 and STAT5 bind to different sites of the IL4 locus. GATA-3 binds to DNaseI hypersensitive site Va and CNS-1 sites of the IL4/IL13 loci, while STAT5 binds to the DNase I hypersensitive sites (HSII and HSIII) in the second intron of the IL4 locus [37, 45].

Recent studies identified the role of STAT3 in Th2 differentiation. STAT3 is required by STAT6 for interaction with relevant gene loci in the developing T cells. It was found that in the absence of STAT3, STAT6 was normally activated, but its interaction with loci was impaired, suggesting the role of STAT3 as a mediator to access to the loci [47, 48]. In STAT3 deficient mice, allergic inflammation was aborted, thereby proving the importance of its presence for the proper development of Th2 cells [47].

IL6, abundantly produced by APCs as well as by nonimmune cells, plays a dual role in Th2 lineage differentiation. It promotes Th2 differentiation, while simultaneously inhibiting the Th1 lineage [49, 50]. The downstream signaling pathway of IL6, in favor of Th2 differentiation, is IL4-dependent. IL6 enhances IL4 production by naïve CD4^+^ cells, through the upregulation of nuclear factor of activated T cells (NFAT). Then IL4 signaling pathway ensures the differentiation as described above. The inhibition of the Th1 development occurs through the IL6-induced upregulation of suppressor of cytokine signaling-1 (SOCS-1) expression, which interferes with STAT1 activation downstream to IFN*γ* signaling [49, 50].

Growth factor independent-1 (Gfi-1) is a transcription repressor, induced by the IL4/STAT6 pathway, as well as by TCR signaling alone. It promotes Th2 cell expansion by selectively enhancing proliferation of GATA3-high cells. In Gfi-1 deficient mice, Th2 cell expansion was significantly reduced [51, 52]. c-Maf selectively upregulates IL4 gene transcription and consequently promotes Th2 cell differentiation by IL4-dependent mechanism [53]. However, c-Maf is not involved in the production of other Th2 cytokines, except for IL4 [46]. Interferon regulatory factor 4 (IRF4) is another transcription factor useful in the lineage specific differentiation of Th2. It coordinates with nuclear factor of activated T cells 2 (NFATc2) to activate IL4 promoter [54]. It has been shown that in the absence of IRF4, IL4 could not induce Th2 differentiation, and GATA3 could not be upregulated despite IL4 treatment. However, the fact that over expression of GATA3 restored Th2 differentiation pathway, one may conclude that IRF4 upregulates GATA3 [55].

### 3.3. Th9 Cells

Initially characterized as a subset of Th2 cells, ongoing researches tend to classify IL9 secreting-Th9 cells as a distinct subset of CD4^+^ T cells. TGF-*β* was found to divert the differentiation of Th2 towards the development of Th9 cells. Moreover, TGF-*β* in combination with IL 4 directly induces the differentiation of Th9 cells [56]. IRF4 also plays an important role. IRF4 was found to directly bind to the IL9 promoter [57]. However, more research need to be conducted to get more insights about the Th9 cells, before being classified as a distinct lineage of CD4^+^ cells.

### 3.4. Th17 Cells Differentiation

IL6, IL21, IL23, and TGF-*β* are the major signaling cytokines involved in Th17 cells differentiation, and retinoic acid receptor-related orphan receptor gamma-T (ROR*γ*t) is the master regulator. The differentiation process can be split into 3 stages, including the differentiation stage mediated by TGF-*β* and IL6, the self-amplification stage by IL21, and the stabilization stage by IL23.

TGF-*β* is the critical signaling cytokine in Th17 differentiation [58–62]. However, TGF-*β* signaling pathways also play significant role in the development of iTreg. Th17 and iTreg are antagonistically related. TGF-*β* alone, at high concentration, can divert lineage differentiation towards iTreg development, through the induction of FOXP3 [63, 64]. However, at low concentration and in the presence of IL6, TGF-*β* induces Th17 differentiation, production of IL21 and upregulates expression of IL23R [58–60, 64]. Since TGF-*β* signaling,unlike IL6, IL21, and IL23, does not activate STAT3, its role appears to involve in the enhancement of STAT3 activation. TGF-*β* inhibits IL6/IL21-induced expression of suppressor of cytokine signaling 3 (SOCS3), which negatively regulates STAT3 signaling pathways [65]. Downstream TGF-*β* signaling pathway in the presence of IL6 leads to the activation of ROR*γ*t [66, 67]. Forced expression of ROR*γ*t induces the production of IL-17A and IL-17F. Besides the master regulator ROR*γ*t, several other transcription factors need to collaborate for full differentiation of Th17 cells. As such, deficiency of ROR*γ*t does not lead to complete interruption of Th17 cytokine expression [67].

STAT3, activated downstream to IL6, IL21, IL23 signaling plays an important role in the differentiation process. It induces ROR*γ*t expression. STAT3 deficiency was found to cause enhanced expression of T-bet and FOXP3, which are involved in the development of opposing cell lineages [68]. STAT3 binds to IL-17A and IL-17F promoters [69].

ROR*α*, another member of the ROR family, also participates in the lineage commitment pathway. Together ROR*α* and ROR*γ*t synergistically enhance Th17 differentiation, and their absence completely aborted the development of Th17 cells [67].

Runx1 also influences Th17 differentiation. Runx1 through the induction of ROR*γ*t, promotes differentiation. However, Runx1/FOXP3 interaction negatively regulates Th17 development [70]. Moreover, T-bet in collaboration with Runx1 leads to the interruption of Runx1-mediated transactivation of Rorc, thereby suppressing Th17 development [71].

Aryl hydrocarbon receptor (AHR), a ligand-dependent transcription factor, was found to promote Th17 differentiation, presumably through the inhibition of STAT1 and STAT5, which negatively regulate Th17 development. However, its absence did not cause complete abortion of Th17 differentiation, but was associated with inability to produce IL22 [72, 73]. Recently identified, activator protein (AP-1) transcription factor, Batf, also plays an important role in the differentiation process. Batf^(−/−)^ mice had defective Th17 response, but Th1 and Th2 development was unaffected [74]. IRF4 was found to be important not only in the differentiation of Th2, but also in that of Th17. Irf4^(−/−)^mice failed to enhance expression of ROR*γ*t and subsequently did not develop experimental autoimmune encephalitis as a result of impaired Th17 response [75]. IRF4 activity is negatively regulated by IRF4 binding protein (IBP), leading to a control of IL17 and IL21 production. Overproduction of the latter cytokines is associated with the development of multiple autoimmune diseases. Mice with deficiency of IBP, rapidly developed rheumatoid arthritis-like joint disease and vasculitis [76].

The self-amplification phase is a crucial step in the differentiation process. It is required in order to mount a robust immune response. Unlike Th1 and Th2 differentiation mechanisms, where their respective major cytokine IFN*γ* and IL4 act as amplifying cytokines, the main cytokine IL17 of Th17 cell does not amplify its differentiation. Instead it is IL21, produced in significant amount by Th17, that in collaboration with TGF-*β* amplify Th17 differentiation. This phase does not require IL6, thereby creating a TCR-independent mechanism of differentiation [77, 78].

The third phase is conducted by IL23, mainly produced by APCs. IL23 is principally required for expansion and maintenance of the Th17 population [58, 79]. IL6 and IL21 downstream signaling induces the expression of IL23R on Th17 cell surface [80]. Moreover IL23 has been shown to induce its own receptor independently [79]. Although thought to be unable to induce Th17 differentiation, recently IL23 in association with IL-1*β* was shown to induce the development of T-bet^+^ROR*γ*t^+^Th17 cells independent of TGF-*β* [81].

### 3.5. Regulatory Cells Differentiation

iTreg cells are FOXP3^+^CD4^+^CD25^+^ cells, which are developed in the peripheral lymphoid organs after antigen priming, in contrast to the natural Treg (nTreg) which are released from the thymus as a distinct lineage with FOXP3 already expressed [82]. TGF-*β* is the critical cytokine responsible for the initiation of the iTreg cell lineage commitment [82–85]. Forkhead transcription factor FOXP3 is specifically expressed in CD4^+^CD25^+^Treg cells and is the major lineage-specific transcription factor involved in iTreg differentiation [85–87]. FOXP3 is induced downstream to TGF-*β* signaling, after interaction with TCR [82, 85]. Fatal immunopathology followed as a result of FOXP3 deletion/mutation, which resulted in defective and decreased iTreg cells [86, 87]. As with the differentiation of the other subsets of CD4^+^ cells, FOXP3 along with other transcription factors is needed for full differentiation of the iTreg cells. 

Smad2 and Smad3, which are also activated through TGF-*β* signaling pathways, are involved in the iTreg differentiation process by inducing FOXP3 [85, 88, 89]. Moreover, Smad 2 and Smad 3 were also found to induce differentiation via FOXP3-independent pathway. Smad3 can differentially enhance iTreg development by upregulating FOXP3 expression and inhibit Th17 differentiation by blocking ROR*γ*t [90].

STAT5-induced downstream to IL2 signaling is required for the differentiation of iTreg [91–94]. STAT5 was found to enhance FOXP3 expression and subsequently downstream to FOXP3 signaling and promote iTreg development. STAT5 and STAT3, which bind to multiple common sites across the IL17 locus, function closely and antagonize each other. Activation of STAT5 by IL2 signaling impair STAT3 binding to the locus sites and consequently enhance iTreg differentiation. Conversely, defective IL2-STAT5 signaling suppresses iTreg, and thus Th17 pathway is favored [91, 95].

NFAT through interaction with FOXP3 promoted Th17 differentiation [89, 96]. Impaired interaction of mutated FOXP3 gene and NFAT led to decreased expression of Treg markers-CTLA4 and CD25 [96].

Among the regulatory cells,Tr1 is being extensively studied. These IL10-producing cells play important role in suppressing inflammation and autoimmune processes. IL27 and IL10 are the principal cytokines involved in driving the Tr1 cells differentiation [97, 98]. IL10 signaling pathways in the induction of the differentiation remains to be elucidated. IL27 signaling leads to the activation of three key factors required for the differentiation. They include the transcription factor c-Maf, IL21, and the costimulatory receptor ICOS. c-Maf is the main factor, whose activation leads to enhanced production of IL21. IL21 acts as an autocrine growth factor driving the expansion of Tr1 cells [99]. ICOS promotes the IL27-induced differentiation of Tr1. Recently, Aryl hydrocarbon receptor (AhR), also induced by IL27, was found to be important in the differentiation of Tr1 cells. AhR and c-Maf act synergistically to mediate the differentiation [100].

### 3.6. Follicular Helper (Tfh) T Cells

Tfh are C-X-C motif receptor-5 (CXCR 5^+^) expressing cells and are located in follicular areas of lymphoid tissue, where they participate in the development of antigen-specific B-cell immunity [101, 102]. IL6 and IL21 are the main cytokines involved in the differentiation process [103, 104]. STAT3, activated downstream to cytokine signaling, is an important transcription factor of Tfh. However, unlike in Th17 development, TGF*β* does not participate, and ROR*γ*t is not induced. In vitro, IL21 in the absence of TGF*β* resulted in Tfh differentiation [105]. Inducible costimulator (ICOS), member of CD28 family, is also required for Tfh development [106, 107]. In mice with ICOSL deficiency, Tfh differentiation was downregulated. More recently Bcl6, a transcription factor selectively expressed in Tfh, was found to play important role in the differentiation. It is activated downstream to IL6 and IL21 signaling, and its overexpression induced Tfh differentiation, while inhibiting opposing cell lineages [108].

## 4. Plasticity of CD4^+^ Cells

**Figure 1:**
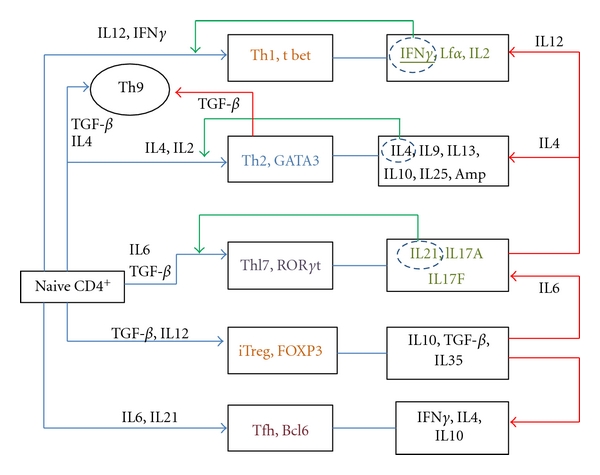
Influence of distinct cytokine milieu in the differentiation of CD4^+^T cells. Blue arrows show the differentiation of naïve cells in the presence of particular cytokines. The green arrows represent the self-amplification phase by the encircled cytokines. Plasticity of T cell subset under the influence of specific cytokine is represented by the red arrows. Along with Th subset, the master regulator is shown. However, Bcl6 has not yet been identified as the master regulator, but it plays major role in the differentiation of Tfh.

Unlike Th1 and Th2 cells, which are considered to be terminally differentiated, Th17 and Treg have shown plasticity, thereby suggesting that they are not terminally differentiated (Figure 1). However, recent studies found that even Th2 cells exhibit plasticity. TGF-*β* caused Th2 cells to switch their characteristic cytokine profile into a IL9 predominating one, suggesting the conversion into Th9 cells [56]. Th17 in the presence of IL12 switched to Th1 phenotype, and interaction with IL4 led to the differentiation into Th2 cells [109, 110]. Treg showed tendency to convert to Th17 and Tfh. In the presence of IL 6, CD4^+^CD25^+^FoxP3^+^ cells upon activation reprogrammed into Th17 [111]. FoxP3^+^Treg in Peyer's patches differentiated into Tfh, with subsequent interaction with B cells and production of Ig A [112]. IRF4 inactivation in Foxp3^+^ cells resulted in Th2 development and increased germinal centre formation [113]. 

## 5. Effector Functions

### 5.1. Th1 Cells

Th1 cells are involved with the elimination of intracellular pathogens and are associated with organ-specific autoimmunity [114]. They mainly secrete IFN*γ*, lymphotoxin *α* (Lf*α*), and IL2. IFN*γ* is essential for the activation of mononuclear phagocytes, including macrophages, microglial cells, thereby resulting in enhanced phagocytic activity [115]. IFN*γ* is believed to exert its effect through the activation of IFN*γ*-responsive genes, which account for more than 200 [116]. One of the well studied is the gene encoding IFN*γ*-inducible GTP-binding protein (IGTP) [105, 117]. IGTP is a member of p47 GTPase family also known as IRG family, is strongly induced by IFN*γ*, and induces the elimination of intracellular pathogens [117, 118]. Lf*α* is a member of the TNF super family. Lf*α* is associated with autoimmune diseases. The depletion of Lf*α* has shown to inhibit the development of experimental autoimmune encephalitis [119, 120]. IL2 promotes proliferation of CD8^+^T cells with acquisition of cytolytic phenotype [121, 122]. Besides its role as T cell growth factor, IL2 was also found to promote the development of CD8^+^ memory cells after antigen priming, and thus participating in ensuring a robust secondary immune response [123]. Natural Treg (thymus derived) need IL2 for survival and activation. Downstream IL2 signaling leads to the activation of STAT5 and eventually to enhanced expression of FOXP3 in naïve cells, thereby acquiring potent suppressive ability [124].

### 5.2. Th2 Cells

Th2 cells mount immune response to extracellular parasites, including helminthes, and play major role in induction and persistence of asthma as well as other allergic diseases [114, 125]. The key effector-cytokines include IL4, IL5, IL9, IL13, IL10, IL25, and amphiregulin. IL4 is a major cytokine involved in allergic inflammation. It is involved in IgE switching and secretion by B cells. IL4 also upregulates low-affinity IgE receptor (Fc*ε*RI) on B-lymphocytes and mononuclear phagocytes, and also high-affinity IgE receptor (Fc*ε*RII) on mast cells and basophils, with subsequent degranulation of the cells and release of several active metabolites, including histamine and serotonin [126]. IL4 also induces the increase of several other proinflammatory mediators, including IL6, GM-CSF (granulocyte-macrophage colony-stimulating factor), VCAM-I adhesion molecule [127]. IL5 mainly targets eosinophils and its precursors, since these cells have relatively higher amounts of IL5R expressed on their surface, and subsequently leads to their activation with upregulation of CD11b and inhibition of apoptosis [128]. IL9 participates actively in the immunopathogenesis of asthma. It activates the function of several cells, including mast cells, B cells, eosinophils, neutrophils as well as airway epithelial cells. Along with hypersecretion of mucus, IL9 was found to release chemoattractant factors, leading to allergic airway inflammation [129]. One of IL13 main roles is to combat gastrointestinal helminthes. IL13, through the activation of cell-mediated immunity, helps in the elimination of intracellular pathogens, such as Leishmania. It also plays a major role in the induction of allergic asthma, through activation of eosinophils, enhanced mucus secretion, and airway hyperresponsivity. Potent stimulation of tissue fibrosis at sites of inflammation was also associated with IL13 [130]. IL10 is an anti-inflammatory cytokine. After pathogen clearance in the course of an immune response, IL10 helps achieve homeostasis through the inhibition of Th1 cells as well as other immune cells of the innate system [131]. IL25, previously known as IL17E, is a member of the IL17 family of cytokines. It is structurally similar to IL17, but functionally different. It promotes Th2 responses [132–134]. It induces increased mucus production, eosinophilia, IgE switching, and enhanced Ig secretion, as a result of upregulation of IL4, IL5, and IL13, thereby amplifying aTh2 response. It was found to induce pathologies of lungs and digestive tract, due to enhanced expression of IL13 [132]. Novel role of IL25 was identified to be the suppression of Th17 response, and consequently the regulation of the development of autoimmune disease. In IL25^(−/−)^ mice, the susceptibility to acquire experimental autoimmune encephalitis was found to be significantly raised, and disease course was accelerated [133]. IL25 suppressed Th17 response by increasing the expression of IL13, which directly inhibit production of cytokines required for development Th17, including IL23, IL1*β*, and IL6 by activated dendritic cells. Moreover, IL25^(−/−)^ mice failed to expel helminthes *Nippostrongylus brasiliensis*, thereby indicating a poor Th2 response [134]. Amphiregulin is a member of the epidermal growth factor (EGF) family. It directly induces epithelial cell proliferation. Its deficiency was associated with delayed expulsion of nematode *Trichuris muris* [135]. The Th9 cell secretes large quantities of IL9, with effects as stated above. At present, Th9 cells are viewed as major culprits in the the development of allergic pathologies, especially asthma [136].

### 5.3. Th17 Cells

Th17 is responsible to mount immune response against extracellular bacteria and fungi. They are also involved in the generation of autoimmune diseases [137–139]. The key effector cytokines include IL17A, IL17F, IL21, and IL22. IL17A and IL17F signaling occurs through a common receptor, IL17RA, thereby suggesting similar functions [140]. Since the receptor IL17RA is expressed in multiple tissues, such as hematopoietic tissue, skin, lung, intestine, and joints, the effect of IL17 extends beyond T cell-mediated inflammatory response. IL17 leads to the induction of proinflammatory cytokines, including IL6, IL1, TNF*α*, and also proinflammatory chemokines ensuring the chemotaxis of inflammatory cells to sites of inflammation [139, 141]. IL21, along being an amplifying cytokine for TH17 development, has pleiotropic functions, including activating T cells, inducing B cells to differentiate into plasmocytes and memory cells, and activating NK cells [142, 143]. IL22 is known to mediate both inflammatory response and exhibits tissue protective properties. IL22 participates actively in mucosal host defense against bacterial pathogens, by inducing antimicrobial peptides and increasing cell proliferation [144]. In acute liver disease, IL22 was shown to be involved in limiting liver tissue damage [145].

### 5.4. Regulatory CD4^+^T Cells

Treg exists as natural thymus-derived subset with expressed FOXP3, and as peripheral-induced Treg cells, which arise from naïve CD4^+^CD25-cells after antigen priming in a relevant cytokine milieu [82]. Treg and Tr1 play important role in the maintenance of immunologic tolerance to self and foreign antigen. After clearance of pathogens, they negatively regulate the immune response, thereby protecting against immunopathology [32, 146]. Their main effector cytokines include IL10, TGF-*β*, and IL35. IL10 is a potent inhibitory cytokine, with the ability to suppress proinflammatory response and thus limits tissue damage by the inflammatory process [131, 147, 148]. IL10 and TGF-*β* potently suppress IgE production, thereby showing their important role in attenuating allergic inflammation [149]. Mice with T-cell-specific deletion of Tgfb1 gene, developed fulminant immunopathology as a result of uncontrolled differentiation of proinflammatory T cells, and hence showing the relevance of TGF-*β* in regulating immune response [150].

### 5.5. Follicular Helper (Tfh) T Cells

After TCR interaction and subsequent differentiation from the CXCR5^−^CCR7^+^CD4^+^ naïve cells, these CXCR5+CD4+T (Tfh) cells play significant role in mediating humoral immunity through interaction with B-lymphocytes. After having lost CCR7, the differentiated CXCR5^+^CCR7-pMHCII-specific Tfh cells enter the pregerminal centre for initial interaction with antigen-primed B cell, with subsequent differentiation of the B cells into Ig-producing plasma cells. In the germinal area, they are involved in the development of long-live B memory cells. According to the predominant cytokine secreted, Tfh cells have been classified into Tfh1, Tfh2, and Tfh10. Tfh1 by secreting IFN*γ* promotes IgG2a production. Tfh2 secretes IL4, which favors the production of IgG1 and IgE. Tfh10 through the secretion of IL10, promotes IgA secretion [151].

## 6. Conclusion

Clearly the CD4^+^T cells represent a unique branch of the adaptive immune system that is crucial in achieving a regulated effective immune response to pathogens, and their proper functioning is vital for survival. Through their distinct phenotypes with their respective cytokine profile, they modulate the functions of the innate immune cells as well as the members of the adaptive immune system. During the recent years, subsets with more specialized and more defined properties have been identified, such as the Tfh and Th9, thereby reinforcing their control over the immune system. Thanks to new technologies, more will be learned about the epigenetic modifications that occur during the differentiation process, and hence we will gain more insights in their development, which will prove useful for later clinical use. Once considered terminally differentiated after antigen-mediated activation, recent studies have been showing the plasticity of the different subsets, particularly the Treg and Th17 cells. This plasticity makes the potential use of Treg risky in autoimmune diseases and organ transplant, since the Treg cells can reprogram into proinflammatory phenotypes in the presence of relevant cytokine milieu and cause more harm. Moreover, aberrantly functioning CD4^+^ cells are associated with the development of multiple autoimmune and allergic pathologies. More research will bring new insights about the epigenetic program of the current and probably novel subsets of CD4^+^T cells and their mechanism and means of functioning, thus subsequently becoming a valuable asset, which clinicians can use against immune-mediated diseases.

## Figures and Tables

**Table 1:** Cytokines and transcription factors (the master regulators are underlined).

CD4^+^ Subset	Cytokines	Transcription factors	Inhibitory transcription factors
Th1	IL12, **IFN*γ***	T bet, STAT1, STAT4, Runx 3, Eomes, Hlx	GATA3
Th2	**IL4**, IL2	GATA3, STAT6, STAT5, STAT3, Gfi-1, c-Maf, IRF4	T-bet, Runx3
Th17	IL6, **IL 21**, IL 23, TGF-*β*	ROR*γ*t, STAT3, ROR*α*, Runx1, Batf, IRF4, AHR	T-bet^+^ Runx1, Smad3 Runx1^+^FOXP3
		
Tfh	IL6, IL21	Bcl6, STAT3	
iTreg	TGF-*β*, IL2	FOXP3, Smad2, Smad3, STAT5, NFAT	
Th9	TGF-*β*, IL4	IRF4	
Tr1	IL27, IL10	c-Maf, AhR	
